# Partially Demineralized Acellular Bovine Bone Matrix Supports for Bone Healing In Vivo

**DOI:** 10.3390/jfb17070330

**Published:** 2026-07-06

**Authors:** Cuc Bui, Quan Minh To, My Thi Ngoc Nguyen, Thuan Minh Le, Triet Minh Tran, Lam Nguyen Le, Duc Hoang Minh Bui, Lam Van Nguyen, Ha Le Bao Tran

**Affiliations:** 1 Faculty of Odonto—Stomatology, Can Tho University of Medicine and Pharmacy, Can Tho 900000, Vietnam; drbuicuc@nhakhoachaua.com (C.B.); lenguyenlam@ctump.edu.vn (L.N.L.); bhmduc@uhsvnu.edu.vn (D.H.M.B.); nvlam@ctump.edu.vn (L.V.N.); 2 Asian Cosmetic Dentistry, Ho Chi Minh City 700000, Vietnam; 3 Faculty of Biology and Biotechnology, University of Science, Ho Chi Minh City 700000, Vietnam; tomquan@hcmus.edu.vn (Q.M.T.); ntnmy@hcmus.edu.vn (M.T.N.N.); 4 Vietnam National University, Ho Chi Minh City 700000, Vietnam; 5 Laboratory of Tissue Engineering and Biomedical Materials, University of Science, Ho Chi Minh City 700000, Vietnam; 6 Cantho Central General Hospital, Can Tho 900000, Vietnam; dr.leminhthuan91@gmail.com (T.M.L.); dr.tmtriet@gmail.com (T.M.T.); 7 Faculty of Medicine, Can Tho University of Medicine and Pharmacy, Can Tho 900000, Vietnam; nvlam@ctump.edu.vn

**Keywords:** decellularization, demineralization, bone matrix, bone healing, osteoconductive, osteoinductive

## Abstract

Acellular bone matrix, with its natural extracellular matrix components, has been considered a potential alternative platform for bone grafting. Our study focused on fabricating acellular bovine bone matrix (ABBM) and evaluating its in vitro characteristics and in vivo effect on bone repair. The bovine cancellous bone was subjected to ABBM preparation, which included partial demineralization and decellularization processing. The effects of the ABBM on human bone marrow-derived stem cells (hBMSCs) were evaluated, including viability, migration, attachment, and proliferation. A rabbit bone defect model was implanted with ABBM and histologically assessed for bone healing. The acellular properties were determined by the absence of nuclear material and the accepted minimum residual DNA content. An in vitro study indicated the ABBM’s positive effect on the migration of hBMSCs. ABBM was also demonstrated to support hBMSC attachment and proliferation. In vivo testing was performed in rabbits with a cranial bone defect, which showed complete bone healing after 8 weeks of grafting with ABBM. Overall, the fabricated ABBM demonstrated in vitro and in vivo biocompatibility and effective support for bone healing in vivo, and therefore represents a potential xenogeneic biomaterial for bone tissue repair.

## 1. Introduction

Bone loss in dentistry occurs immediately after tooth loss and lasts for many years afterward, leading to a significant reduction in bone volume if teeth are lost for a long time. The consequence of this phenomenon is that the jawbone becomes weaker, changing the position of surrounding teeth and affecting the patient’s esthetics [1,2]. Guided bone regeneration (GBR) is a technique that uses bone substitute materials to graft into the defect site, forming a “skeleton” for bone tissue cells to come and create new bone. Meanwhile, the material mass will gradually be absorbed, making room for newly formed bone tissue [3,4]. Therefore, the GBR has been considered a broad indication for bone defect treatment because it is not limited by the volume of bone tissue to be removed and can be flexibly combined with other methods. Autologous bone grafting is considered the gold standard in the GBR technique. Autologous bone has bone-stimulating properties and good bone compatibility, but is quickly resorbed. Autologous bone grafting is more favorable for minor bone defects due to limited quantities of harvest. Therefore, bone sources other than autologous bone are widely used in GBR surgery [4].

Bovine bone has bone-conductive properties, good bone compatibility, and a long lifespan in the body, therefore it gains research attention for fabrication into bone grafts. There are currently two approaches in bovine bone processing with distinct techniques and outcomes, including deproteinization and decellularization. Both strategies share a similar objective in minimizing the immunogenicity of the xenogenic bone grafts. Deproteinization aims to eliminate all the protein components in the bone matrix, while preserving the mineral components. One of the well-known deproteinized bone grafts available on worldwide market is Bio-Oss (Geistlich Biomaterials, Princeton, NJ, USA). The deproteinized bone grafts exhibit osteoconductivity; however, they lack an intrinsic property of osteoinductivity [5,6,7]. Therefore, in bone tissue engineering research and clinical practice, the deproteinized bone grafts have been combined with bone growth factors (BMP-2, e.g., [7,8]), osteogenic progenitor cells/mesenchymal stem cells [2,9], platelet-rich plasma/fibrin [10,11] or mixed with autogenous bone graft with a proposed 50:50 ratio [12,13,14].

Decellularization focuses on the removal of cellular components using various physical, chemical, and enzymatic processing techniques. The extracellular matrix (ECM) structure of bone tissue can be preserved by optimizing decellularization agents. The ECM components, such as collagen and bioactive proteins associated with bone ECM, can effectively promote bone healing. It was found that decellularized bovine bone possessed a high content of BMP-2 and BMP-7, with preserved fibrous collagen microarchitecture [15]. Decellularized bovine bone also facilitated growth and osteogenic differentiation in vitro. New bone formation was detected in mouse calvarial defect treated with decellularized bovine bone. Bovine cancellous bone scaffolds were also demonstrated to facilitate human osteoblast cell attachment, proliferation, and mineralization activity, suggesting that the decellularized bone scaffold with minimal ECM damage could perform osteoconduction, osteoinduction, and osteogenesis in vitro [15,16,17]. Therefore, the decellularized bone matrix has been considered as a potential alternative platform for bone grafting with effective osteoconductivity compared with deproteinized bone grafts.

In the approach of cellular removal, there are two typical processes to be proposed: demineralization and decellularization. The structural characteristics of bovine trabecular bone tissue contain type I collagen, mineralized with calcium phosphate—mainly hydroxyapatite. During processing, this highly compacted mineralization also serves as strong physical barrier, hindering the effect of treatment reagents. Therefore, demineralization not only provides decellularization aid but also exposes the remaining ECM and embedded bioactive factors. Hydrochloric acid (HCl) at 0.5–0.6 N is commonly utilized for mineral extraction of bone tissue, at durations up to 24 h, resulting in a soft bone matrix [16,18,19] with a significant decrease in mineral content (e.g., 0.1% Calcium compared to 41.8% in native tissue control). Indeed, demineralized bone matrix has been used in dentistry as extrudable material [20], or further fabricated into hydrogel in bone tissue engineering research [18,19]. Recently, partially demineralized allogeneic bone grafts, achieved via 1 h–1 h 30 incubation in 0.4 N HCl, in combination with chemical treatment and autoclaving, achieved sterilization of the grafts. The 7-week in vivo testing revealed appropriate bone healing in rat calvaria [21,22]. The heat treatment was indicated as a potential cause of growth factor denaturation and, therefore, needed further consideration to conserve the bone matrix bioactivity.

Our previous publication employed a 15 min treatment in 0.6 N HCl in the decellularization procedure of porcine bone tissue, which showed good in vitro and in vivo biocompatibility [23]. This short-term HCl incubation was also applied on bovine bone tissue for partial demineralization, followed by methanol-chloroform for 6 h and 0.15% SDS for 24 h, and presented complete cell removal [24]. Meanwhile, the partial demineralization of bovine bone tissue has not been mentioned elsewhere; our proposed procedure would introduce an alternative approach in preparing acellular bovine bone matrix for bone defect repair. In this study, the partially demineralized and decellularized bone particles were fabricated and investigated for their biocompatibility by means of supporting the attachment, proliferation, and migration of human bone marrow-derived stem cells. The bone-healing effect was evaluated in vivo via rabbit calvarial defect intervention.

## 2. Materials and Methods

### 2.1. Materials

Femurs of adult Sindhi crossbred cattle weighing 400–450 kg were collected after slaughter at the slaughterhouse and transported to the laboratory on ice. Human bone marrow-derived stem cells (hBMSCs) were provided by the Laboratory of Tissue Engineering and Biomedical Materials, University of Science, VNU-HCM. Mouse fibroblasts (L929, Accession number 70026472) were purchased from ATCC, USA. Basal medium was defined as DMEM-F12 (Sigma-Aldrich, St. Louis, MO, USA) supplemented with 1% Penicillin-Streptomycin antibiotics (Sigma-Aldrich, St. Louis, MO, USA). L929 and hBMSCs were cultured at 37 °C, 5% CO_2_ in complete medium comprising basal medium and 10% fetal bovine serum (Sigma-Aldrich, St. Louis, MO, USA).

### 2.2. Acellular Bovine Bone Matrix (ABBM) Preparation

The bovine cancellous bone was obtained mechanically. The cancellous bone was cut into 0.5 × 0.5 × 1 cm blocks and washed with saline to completely remove bone fragments and other parts such as blood, fat and muscle. Bovine cancellous bone underwent the decellularization process according to our previous investigation. Briefly, the bone specimens were treated in methanol/chloroform solution (ratio 1:1) for 6 h, followed by demineralization in 0.6 M HCl for 15 min. Decellularization was conducted by using 0.15% sodium dodecyl sulfate (SDS) (Sigma-Aldrich, St. Louis, MO, USA) for 24 h [24]. The treated specimens were washed in distilled water for 48 h at room temperature with agitation at 80 rpm. The acellular properties were determined by sample DNA quantification, histological evaluation, including Hematoxylin and Eosin (H&E) staining, and DAPI staining. The ABBM specimens were freeze-dried, homogenized into 1–2 mm particles, and sterilized by 25 kGy gamma irradiation.

### 2.3. In Vitro Cytotoxicity Evaluation via ABBM Liquid Extract

ABBM liquid extract was prepared by incubating the sample particles in complete medium at 37 °C for 24 h (0.2 g per mL, according to ISO 109930-12 [25]). Complete medium and 20% DMSO in complete medium were utilized as negative and positive controls, respectively. L929 cells were seeded into a 96-well plate (10^4^ cells per well) and cultured at 37 °C for further attachment. The cells were then incubated with either the ABBM liquid extract or controls for 24 h. MTT assay was conducted for cell viability assessment, including 4 h cell incubation with 0.5 mg/mL MTT solution (Sigma-Aldrich, St. Louis, MO, USA) and optical absorbance measurement at 570 nm (EZ-400, Biochrom, Cambridge, UK). The relative growth rate (RGR—%) was calculated as OD570 nm (test group)/OD570 nm (negative control) x 100%. The non-toxic effect of ABBM liquid extract was confirmed when the RGR value exceeded 70% [26].

### 2.4. Scratch Wound Healing Assay for Investigating hBMSC Migration Under ABBM Liquid Conditions

hBMSCs were seeded into a 35 mm culture dish (5 × 10^4^ cells) and cultured at 37 °C for further attachment and to form a confluent cell monolayer. Before the assay, the cells were starved for 24 h by treating with DMEM-F12 basal medium. ABBM liquid condition was prepared by incubating ABBM particles in basal medium at 37 °C for 24 h. On the day of the experiment, a straight scratch was generated onto the cell monolayer using a sterile 200 µL pipette tip. After cleansing for cell debris removal, hBMSCs were treated with the ABBM liquid condition for 24 h. Basal and complete medium were used as negative and positive controls, respectively. Cell migration into the acellular scratch was monitored at 0 and 24 h timepoints, and figures were captured with an Olympus CKX-53 microscope (Olympus, Tokyo, Japan) equipped with a digital camera (Olympus, Tokyo, Japan). The scratch areas from three images from each group (*n* = *3*) were analyzed by ImageJ software (ImageJ 1.54g, National Institudes of Health, USA), followed by calculation into percentage (%) of remaining acellular area using (scratch area at 24 h timepoint/scratch area at 0 h timepoint) × 100%.

### 2.5. hBMSC Attachment on ABBM

ABBM particles were placed into a 96-well plate and incubated in complete medium overnight. hBMSCs were seeded into a 96-well plate containing ABBM particles (2 × 10^4^ cells per well) and cultured at 37 °C for 48 h. Cell viability and attachment on ABBM scaffolds were examined by Calcein assay and Scanning Electron Microscope observation (SEM). The cell-seeded specimens were incubated in Calcein solution (ratio 1:1000, Thermo Scientific, Waltham, MA, USA) for 45 min. Viable cell attachment on ABBM was observed under a fluorescence microscope with a green filter (Olympus, Tokyo, Japan). The cell-seeded specimens were also fixed in SEM solution for 24 h, followed by dehydration using increasing ethanol concentrations of 30%, 50%, 70%, 80%, 90%, and 100%. SEM analysis was performed using JSM-6510 (JEOL, Tokyo, Japan).

### 2.6. hBMSC Proliferation on ABBM

ABBM particles were placed into a 96-well plate and incubated in complete medium overnight (5–6 particles in each well). hBMSCs were seeded into a 96-well plate containing ABBM particles (10^4^ cells per well) and cultured at 37 °C for 24 h. The cell-seeded specimens were transferred into a new sterile 96-well plate using sterile forceps for further culture and proliferation assessment. Cell Counting Kit-8 (CCK-8) was applied to monitor cell proliferation on ABBM. On the assay day, the cell-seeded specimens were incubated with 100 μL of complete medium containing 10% CCK-8 solution (Sigma-Aldrich, St. Louis, MO, USA). After 4 h, the supernatant in each tested well was harvested for optical absorbance measurement at 450 nm (EZ-400, Biochrom, Cambridge, UK). The specimens remained in the wells and were cultured in complete medium for the necessary timepoints, including day 4, 7, and 10.

### 2.7. In Vivo Animal Test for Bone Healing Support

Adult male New Zealand White rabbits, each weighing 3 kg (±250 g), were used in this study with the approval of the Animal Care and Use Committee of the University of Science (Approved number: 1493/KHTN-ACUCUS). Animals were provided with an appropriate balanced dry diet and water ad libitum, and were caged individually. All animals were allowed seven days from their arrival at the facility to acclimatize to their new environment.

This study investigated calvarial defect regeneration in rabbits, including three groups: Group BLK served as the control with no therapeutic treatment, Group ABG received autologous bone grafting, Group ABBM was treated with the fabricated bone matrix. Three animals were used in this pilot investigation (*n* = 3) [19,21]. After an 8-week healing period, the animals were targeted for harvesting grafted specimens. An intramuscular injection of 5 mg/kg Xylazine (TROY, New South Wales, Australia) and 15 mg/kg Zoletyl dose 50 (Virbac, Westlake, TX, USA) was used to anesthetize the animals. The calvaria bones containing the healed sites were surgically harvested and immediately fixed in neutral buffered formalin (10%) for 24 h, followed by histological process and Hematoxylin and Eosin staining. The tissue sections were observed and captured using a light microscope (Nikon, Tokyo, Japan) equipped with digital microscope camera (Nikon, Tokyo, Japan).

### 2.8. Statistical Analysis

All data are presented as means ± standard deviation of three replicates. Statistical analysis was performed for each experimental group using one-way ANOVA and GraphPad Prism 10 (GraphPad Software, Inc., San Diego, CA, USA). A *p*-value of *p* < 0.05 was considered a significant difference.

## 3. Results

### 3.1. Characterization of Acellular Bovine Bone Matrix

**Figure 1:**
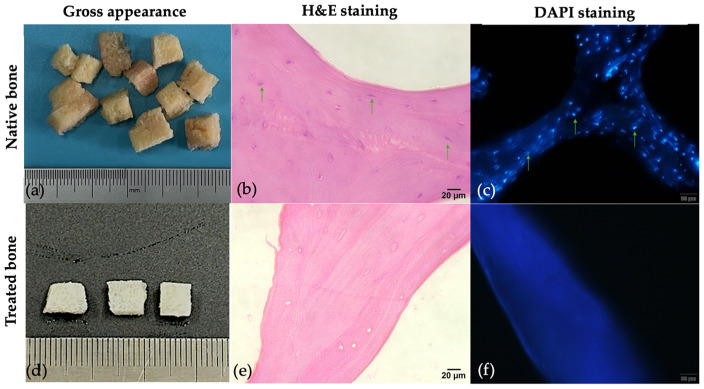
Gross appearance and histological examination of native and treated bone. (**a**) Native bone specimens. (**b**,**c**) H&E and DAPI staining of native bone [24]. (**d**) Treated bone specimens. (**e**,**f**) H&E and DAPI staining of treated bone [24]. The arrows indicate the cell nuclei. The scale bar is 20 μm (H&E images, 400× magnification) and 50 μm (DAPI images, 200× magnification).

**Figure 2:**
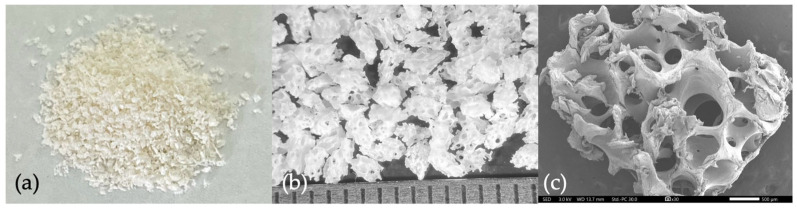
ABBM particle preparation and morphology evaluation. (**a**) ABBM particles. (**b**) ABBM particle observed via a stereo microscope [24]. (**c**) Representative SEM image of an ABBM particle. The scale bar is 500 μm (SEM images, 30× magnification).

The bone specimens contained fat and blood with a yellowish-red color, and presented the native cellular components as detected nuclei within the tissue matrix (Figure 1a–c). After processing, the bone specimens appeared white and clean, indicating the natural color of bone extracellular matrix (Figure 1d). H&E and DAPI staining demonstrated no presence of the cellular nuclei components (Figure 1e,f). Following the decellularization procedure, the DNA remnants were significantly reduced to less than 50 ng per mg dry weight (38.4 ± 6.5 ng/mg, *n* = 3). Taken together, the treated bone samples were considered to meet the minimal acellular criteria; therefore, they were determined as acellular bovine bone matrix (ABBM). The ABBM specimens were freeze-dried, homogenized into 1–2 mm particles for further experiments (Figure 2a). Both macroscopic and SEM evaluation revealed a porous structure of ABBM with 100–500 μm pore size (Figure 2b,c).

### 3.2. In Vitro Cytotoxicity of ABBM Liquid Extract

**Figure 3:**
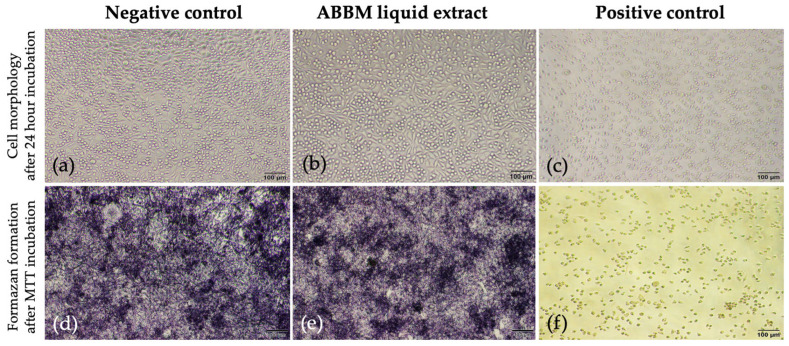
In vitro cytotoxicity assay on L929 cells [24]. (**a**,**d**) Negative control as complete medium. (**b**,**e**) ABBM liquid extract. (**c**,**f**) Positive control as 20% DMSO in complete medium. The scale bar is 100 μm (100× magnification).

ABBM liquid extract was demonstrated to induce no cytotoxicity on L929. Upon incubation in the complete medium and ABBM liquid extract (Figure 3a,b), L929 cells remained attached to the culture dish and exhibited their normal morphology. Meanwhile, cell death with a rounded morphology and detachment was detected after 24 h of treating with the 20% DMSO medium (Figure 3c). Correspondingly, a dense formation of formazan crystals in the L929 cell layer treated with complete medium and ABBM liquid extract (Figure 3d,e), which was absent in the positive control (Figure 3f). ABBM liquid extract-treated L929 reached a RGR value as 90.9 ± 2.0% (*n* = 3), significantly higher than 70% as a non-toxicity criterion.

### 3.3. hBMSC Migration Under ABBM Liquid Condition

**Figure 4:**
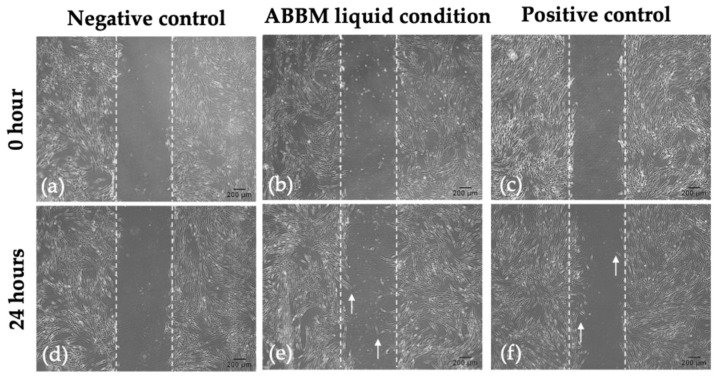
Scratch wound healing assay for hBMSC migration evaluation. (**a**,**d**) Negative control as basal medium. (**b**,**e**) ABBM liquid condition. (**c**,**f**) Positive control as complete medium. The scale bar is 200 μm (40× magnification). White arrows indicate the cells that migrated into the scratch area. The white dashed lines indicate the scratched area.

**Figure 5:**
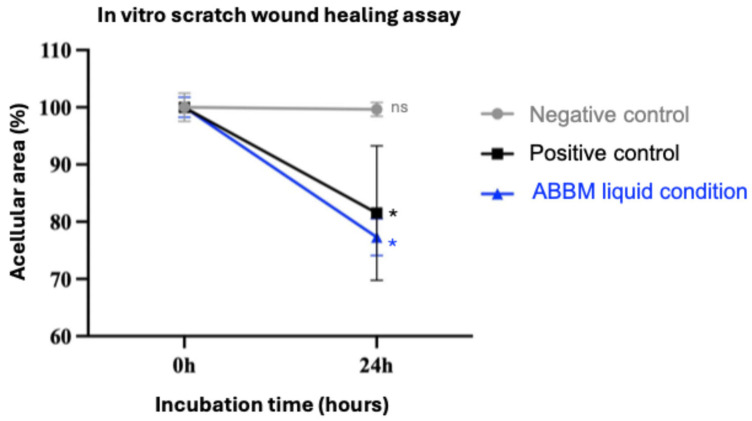
The change in acellular area (%) after cell migration under different conditions. Gray line indicates basal medium as negative control. Black line indicates complete medium as positive control. Blue line indicates the liquid condition extracted from ABBM. *: statistical difference (*p* value < 0.05); ns: no statistical difference (*p* value > 0.05); the two-sample *t*-test is used to compare the change in acellular area between two incubation time points.

The effect of ABBM liquid condition on hBMSC migration was investigated using a scratch wound healing assay for 24 h (Figure 4). Basal and complete medium were used as negative and positive controls, respectively. At 0 h, an artificial acellular area was generated on the cell monolayer by the scratching effect (Figure 4a–c). After culturing for the next 24 h, there was no cell migration in the negative control (Figure 4d). The presence of hBMSCs was observed in the scratch area under ABBM liquid conditions (Figure 4e), as was similarly observed in the positive control (Figure 4f). In both the positive control and the conditions with ABBM liquid extract, the acellular area was found to reduce after 24 h incubation (Figure 5, *p* value < 0.05). This result suggested that ABBM liquid conditions support migration toward hBMSCs.

### 3.4. hBMSC Attachment on ABBM Scaffolds

**Figure 6:**
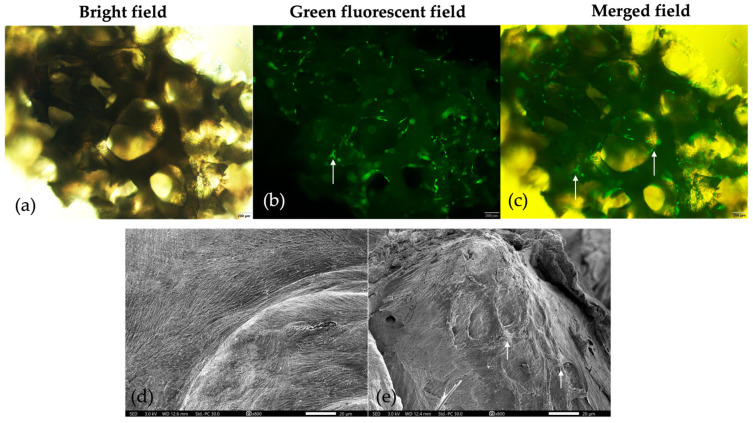
hBMSC attachment on ABBM scaffold. (**a**–**c**) Calcein incubation and detection by fluorescent microscope. The scale bar is 200 μm (40× magnification). (**d**,**e**) SEM examination of ABBM scaffold and cell-seeded ABBM scaffold. The scale bar is 20 μm (800× magnification). White arrows indicate cells adhered to the ABBM scaffold.

In fluorescence observation after calcein incubation, the result illustrated hBMSC attachment on the surface of ABBM scaffold (Figure 6a,b, white arrows). Live cells were indicated in green fluorescence excitation (calcein AM), which distributed evenly throughout the porous scaffold (Figure 6c, white arrows). Regarding SEM evaluation, the image revealed a blank surface of ABBM scaffold without cell seeding (Figure 6d). In the hBMSC-seeded scaffold, SEM imaging indicated the presence of hBMSC attachment with a spreading appearance on the scaffold (Figure 6e, white arrows).

### 3.5. hBMSCs Proliferation on ABBM Scaffolds

**Figure 7:**
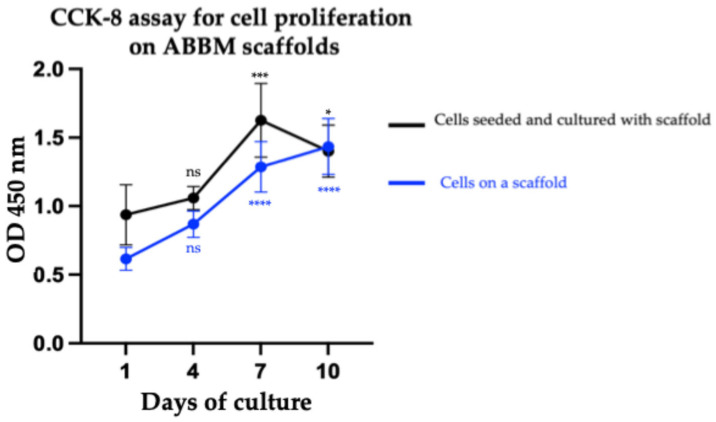
CCK8 assay for hBMSC proliferation on ABBM scaffold. Blue line indicates the cell-seeded scaffold group which were cultured separately from the initial plating wells. The black line indicates the setup, where ABBM scaffolds are seeded with hBMSC suspension and subjected to a prolonged culture duration. ns: not significant, *p* value > 0.05; *: *p* value < 0.05; ***: *p* value < 0.001; ****: *p* value < 0.0001; one-way ANOVA with Tukey’s post hoc test, where day 4, day 7, and day 10 were compared the initial timepoint.

The effect of ABBM scaffold on hBMSC proliferation was investigated by CCK-8 assay for 1–10 days. The cell-seeded scaffolds were separated from the initial plating wells and monitored for proliferation (Figure 7, blue line), which represented for the cell proliferation on ABBM scaffold. An increase in OD450 values was observed over 10 days of culture, with a statistically significant difference on day 7 (*p* value < 0.0001) and day 10 (*p* value < 0.0001) compared to the initial timepoint, suggesting the supportive effect of the ABBM scaffold in hBMSC proliferation.

Meanwhile, a different cell proliferation assessment was also conducted, in which ABBM scaffolds were seeded with hBMSC suspension and prolonged culture duration (Figure 7, black line). This experimental group aimed to evaluate hBMSC proliferation on both the culture surface and ABBM scaffold. It also observed the cell growth throughout the 10 days, with the basal OD450 values higher than that in the cell-seeded scaffolds. This comparison indicated hBMSCs performed an appropriate proliferation on both the ABBM scaffold and on the culture dish surface in the presence of ABBM.

### 3.6. ABBM Grafts Supported Bone Healing In Vivo

All the animals recovered after 3 days of material implantation. There were no signs of health problems, such as fever, excessive sleepiness, or loss of appetite. After 7–10 days, the sutured sites were completely healed, with no signs of inflammation. Observations after eight weeks showed that the rabbits had no signs of inflammation at the implanted site of their heads, their health was stable, and their daily activities remained normal. The implants were surgically exposed to assess bone-healing outcomes.

**Figure 8:**
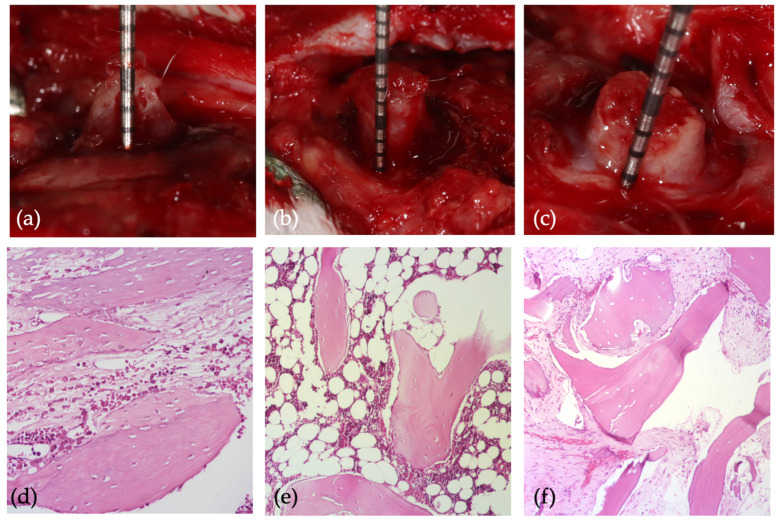
ABBM grafts supported bone healing in vivo. (**a**–**c**) Macroscopic observation of implanted site. (**d**–**f**) Histological assessment of the implanted tissues. (**a**,**d**) Un-grafted group as BLK. (**b**,**e**) Autologous bone grafted group as ABG. (**c**,**f**) Acellular bovine bone matrix grafted group as ABBM. (100× magnification).

**Figure 9:**
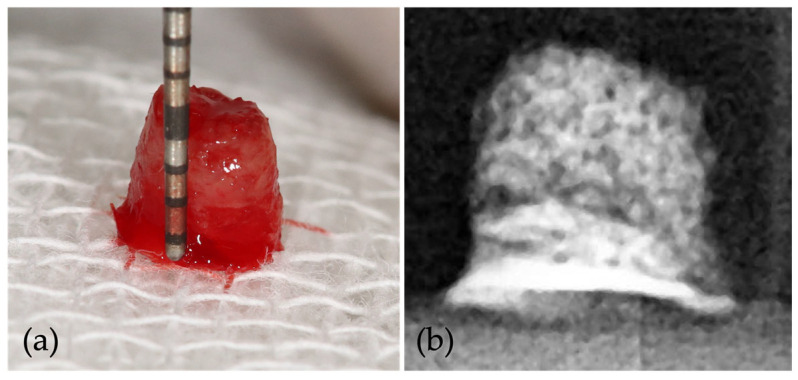
X-Ray image of the implanted ABBM after eight weeks. (**a**) Gross observation. (**b**) X-Ray image of the implanted ABBM.

The macroscopic figures indicate the longitudinal measurement of new bone formation in rabbits over an 8-week period (Figure 8a–c). Figure 8d–f present histological sections of calvarial defects stained with Hematoxylin and Eosin across three experimental groups, including the BLK, ABG, ABBM. In the macroscopic observation, Group BLK revealed a minimal new bone formation (Figure 8a). Group ABG (Figure 8b) and ABBM (Figure 8c) both showed greater bone formation. Histological examination demonstrated that the grafted ABBM particles were surrounded by connective tissue (Figure 8f) without significant inflammatory response, showing good biocompatibility. The X-Ray examination of group ABBM (Figure 9b) showed the implanted area with a cancellous-like morphology, indicating a bone tissue healing response.

## 4. Discussion

The abundant availability of xenogeneic tissues makes bovine bone an attractive source for scaffolds and bone graft fabrication to meet the growing demand for bone grafts in dentistry and transplantation. In this study, we aimed to assess the bone-healing potency of acellular bovine bone matrix using human stem cells in vitro and a rabbit model in vivo.

The acellular bovine bone matrix (ABBM) was produced by using extraction techniques, including lipid removal, short-term demineralization, and decellularization. The cancellous bone in the bovine femur contains a high level of lipid quantity, which can form a water-in-oil emulsion and hinder the absorption of the reagent–water mixture. Therefore, delipitation was carried out in the first instance using a mixture of methanol and chloroform, which effectively dissolves lipids and lipid-derived cellular components [23,27]. Our preliminary research also included partial demineralization via HCl incubation for 15 min as part of the decellularization procedure. Demineralization was shown to positively promote the release of ECM bioactive factors that are beneficial to bone healing effectiveness [20,28]. The demineralization can be carried out by immersing the bone sample in acid solutions. HCl at 0.5–0.6 N is frequently used for demineralization of bone samples due to its effective reaction with the calcium-containing mineral complex in bone tissue. The 24- to 48 h HCl treatment has been used in bone tissue decellularization, resulting in a soft matrix that facilitates the permeability of the chemical detergent for cellular removal [16,19,29]. In contrast, the decreased level of inorganic components significantly alters the mechanical strength and accelerates bone graft resorption. Therefore, in the utilization of xenogenic bone tissue for bone grafting, the balance between introducing the biological bone ECM with sufficient inorganic content necessitates an appropriate combination of demineralization and decellularization. Recently, a 1 h treatment with HCl was utilized for allogenic bone powder (0.3–1 mm in diameter) and was considered as partial demineralization [21,22], which presented adequate bone regeneration compared to syngeneic bone graft in vivo rat models. The shorter duration of HCl treatment, indicated as 15 min, has been applied to human dentin tissue and is noticeable as partial demineralization. It is also indicated that brief exposure to HCl, such as 15–30 min for demineralization, was selectively employed in the clinical indication rather than for material research on bone graft fabrication [30]. The partial demineralization of bovine bone tissue has not been previously reported; therefore, our method offers an alternative approach for preparing ABBM for use in bone defect repair.

Decellularization of xenogeneic cellular antigens was carried out by immersing them in 0.15% Sodium dodecyl sulfate (SDS) for 24 h. SDS is commonly used for tissue decellularization due to its capability to lyse the cells efficiently. Upon partial demineralization of bone matrix, 0.15% SDS was also effective for decellularization and can be considered to meet the minimum standards for immune response in medical applications. The SDS concentration was considered milder than that used in previously published protocols, which ranged from 0.5 to 1% SDS [16,29]. Therefore, it was proposed that the fabricated ABBM would preserve ECM components and positively affect bone healing. Indeed, the potential effect of ABBM was demonstrated by appropriate support for hBMSC wound-healing behavior in vitro. The ABBM liquid extract was shown to guide hBMSC migration, thereby recruiting stem cells to the site of bone repair [31]. The ABBM offered a supportive scaffold for hBMSC attachment and proliferation, indicating its integration capacity with native bone tissue.

However, to broadly elucidate the bioactivity of the fabricated ABBM, further investigation is needed. The growth factor secretion profile of ABBM, including TGF-β and BMPs, supports the positive effect of ABBM on hBMSC migration. To determine the level of hBMSC migration support by ABBM extract, a quantitative examination, including a transwell assay, could be a potential approach. In vitro osteogenic differentiation would be suggested to determine the osteoinductive properties of ABBM and to provide an in vitro basis for in vivo bone healing after ABBM grafts. In addition, the mineral content, including calcium and phosphate, and mechanical strength and stiffness, would be taken into account to provide a full understanding of the biomechanical properties of the fabricated ABBM. In the fabrication procedure, the sterile condition of the grafts is one of the most important criteria regarding the product’s biosafety. A dose of 25 kGy or higher is considered a standard radiation sterilization dose for medical products, including bone tissue-derived products [16,32]. Therefore, 25 kGy gamma irradiation was applied to ABBM to ensure sterile conditions before conducting the cell assays. However, to maintain ECM integrity and bioactive components, the dose dependence of gamma irradiation and the effects of excessive manipulation, including dehydration and storage conditions, also require more elucidation, in addition to biosafety considerations such as endotoxin and pyrogenicity for potential implantation use.

The animal study was to evaluate the host response after implantation of ABBM particles in rabbits with examined bone defects. It was recorded that no fibrosis developed between the particles of the ABBM and the regenerated bone. Similar to the autologous bone grafting, there were also no inflammatory responses observed for the ABBM intervention, suggesting its biocompatibility in vivo. New bone formation was observed in the ABBM grafting, as indicated by an increase in bone size and height compared with the un-grafted group. This pilot examination of bone-healing effect was primarily assessed by histological examination and X-Ray images. The next investigation would focus more on details, such as bone density and microstructural integrity, using micro-CT and immunohistochemistry. Given that the preliminary results on the bone-healing effect are based on a relatively small number of animals, further studies involving larger animal cohorts are proposed to support more comprehensive and statistical analyses of ABBM’s effect on bone regeneration.

## 5. Conclusions

The study introduced partial demineralization and decellularization of bovine bone to prepare an acellular material. The liquid extract of the material was shown to support mesenchymal stem cell migration in vitro, suggesting its ability to provide biomimetic cues for bone-healing outcomes. The acellular bone matrix also provides a biocompatible scaffold for cell attachment and proliferation in vitro. In vivo testing in rabbit cranial defects confirmed its biocompatibility and facilitated bone healing after eight weeks. These findings highlight the potential of acellular bovine bone matrix to support bone-healing therapy. Regarding the challenge of adopting xenogenic bone tissue for clinical use, there is a need to elucidate the detailed characteristics of the ABBM to support bone tissue repair applications in the near future.

## Data Availability

The original contributions presented in this study are included in the article. Further inquiries can be directed to the corresponding author.

## Abbreviations

The following abbreviations are used in this manuscript:

ABBM	Acellular bovine bone matrix
hBMSC	Human bone marrow-derived mesenchymal stem cell
BLK	The animal control group with no therapeutic treatment
ABG	The animal group received autologous bone grafting
